# Activated NAD^+^ biosynthesis pathway induces olaparib resistance in *BRCA1* knockout pancreatic cancer cells

**DOI:** 10.1371/journal.pone.0302130

**Published:** 2024-04-16

**Authors:** Yuka Sasaki, Takuma Inouchi, Ryusuke Nakatsuka, Amane Inoue, Mitsuko Masutani, Tadashige Nozaki

**Affiliations:** 1 Department of Pharmacology, Faculty of Dentistry, Osaka Dental University, Hirakata, Osaka, Japan; 2 Department of Molecular and Genomic Biomedicine, Center for Bioinformatics and Molecular Medicine, Nagasaki University Graduate School of Biomedical Sciences, Sakamoto, Nagasaki, Japan; Qatar Biomedical Research Institute, QATAR

## Abstract

PARP inhibitors have been developed as anti-cancer agents based on synthetic lethality in homologous recombination deficient cancer cells. However, resistance to PARP inhibitors such as olaparib remains a problem in clinical use, and the mechanisms of resistance are not fully understood. To investigate mechanisms of PARP inhibitor resistance, we established a *BRCA1* knockout clone derived from the pancreatic cancer MIA PaCa-2 cells, which we termed C1 cells, and subsequently isolated an olaparib-resistant C1/OLA cells. We then performed RNA-sequencing and pathway analysis on olaparib-treated C1 and C1/OLA cells. Our results revealed activation of cell signaling pathway related to NAD^+^ metabolism in the olaparib-resistant C1/OLA cells, with increased expression of genes encoding the NAD^+^ biosynthetic enzymes NAMPT and NMNAT2. Moreover, intracellular NAD^+^ levels were significantly higher in C1/OLA cells than in the non-olaparib-resistant C1 cells. Upregulation of intracellular NAD^+^ levels by the addition of nicotinamide also induced resistance to olaparib and talazoparib in C1 cells. Taken together, our findings suggest that upregulation of intracellular NAD^+^ is one of the factors underlying the acquisition of PARP inhibitor resistance.

## Introduction

Poly ADP-ribosylation (PARylation) is a post-translational modification that contributes to various biological functions, including DNA repair of single-strand breaks (SSBs) and double-strand breaks (DSBs), transcriptional regulation, histone modification, and cell death [1–3]. This reaction is catalyzed by poly (ADP-ribose) polymerase (PARP) family proteins such as PARP1 and PARP2, which use β-nicotinamide adenine dinucleotide (NAD^+^) as a substrate to produce long ADP-ribose chains that are then added to target proteins [4, 5]. Degradation of poly (ADP-ribose) to ADP-ribose by the enzymes poly (ADP-ribose) glycohydrolase (PARG) and ADP-ribosyl hydrolase 3 regulates the various functions affected by PARylation [6–8].

PARP inhibitors that target PARP1/2, such as olaparib and talazoparib, have been approved as anticancer agents for patients with breast and ovarian cancers bearing mutations in the homologous recombination (HR) factors *BRCA1* and *BRCA2*, as well as for platinum-sensitive cancers [9–12]. Mechanistically, PARP inhibitors are thought to act via inhibition of the lethal DNA repair pathways (SSB repair and non-homologous end joining repair) and are therefore effective against tumors deficient in HR repair as a result of defects in *BRCA1* or *BRCA2* [13]. Thus, PARP inhibitors based on the concept of synthetic lethality are expected to have few side effects because these drugs induce cell death in HR repair-deficient cancer cells but not in normal, HR-proficient cells. Among various types of cancers, the prognosis for pancreatic cancer patients is particularly poor, with a 5-year survival rate of about 10% [14], and no effective definitive therapy has so far been developed. Recently, olaparib was approved for patients with *BRCA*-mutated pancreatic cancer, estimated at 4% to 7% of total patients with pancreatic cancers [15]. The Pancreas Cancer Olaparib Ongoing (POLO) trial reported that progression-free survival was significantly longer in the olaparib-treated group than in the placebo group [15], indicating that olaparib is effective in *BRCA*-mutated pancreatic cancer patients. Nonetheless, although the therapeutic effects of PARP inhibitors have been reported, the acquisition of resistance to PARP inhibitors by continuous administration remains a therapeutic problem.

Recently, resistance factors for various PARP inhibitors have been reported in cancer patients and tumor cells. For example, some types of reversion mutations of *BRCA1* and *BRCA2* are associated with acquired resistance to PARP inhibitors, including olaparib, in *BRCA1*/*2*-mutated tumors and cell lines [16]. As another resistance mechanism, loss of either 53BP1 or the Shieldin complex including REV7, SHLD1 and SHLD2 in NHEJ pathway induced resistance to PARP inhibitors via restoration of HR in *BRCA1* deficient cells [17, 18]. PARP inhibitors have also been reported as substrates of the drug efflux pump MDR1, and overexpression of MDR1 induced resistance to PARP inhibitors [19]. Further mechanisms of PARP inhibitor resistance in *BRCA*-mutated cancer cells include decreased PARP1 trapping by *PARP1* mutation [20] and downregulation of PARG [21], and replication fork stabilization by loss of EZH2 and PTIP [22]. Despite these findings, the mechanisms of PARP inhibitor resistance are not fully understood, and it remains unclear how to overcome resistance to PARP inhibitors.

In the present study, we established a *BRCA1* knockout (KO) pancreatic cancer cells and subsequently isolated an olaparib-resistant clone from these cells. To analyze novel mechanisms of resistance to PARP inhibitors, we carried out RNA-sequencing (RNA-seq) analysis and pathway analysis to compare olaparib-treated *BRCA1* KO cells and olaparib-resistant cells. Our results suggest that increased intracellular NAD^+^ levels are involved in the induction of PARP inhibitor resistance.

## Material and methods

### Cell culture and reagents

The human pancreatic cancer cell line MIA PaCa-2 and C1 cells established in this study were cultured in minimal essential medium (MEM)(Nacalai Tesque, Kyoto, Japan) supplemented with 10% fetal bovine serum (FBS) (Cosmobio, Tokyo, Japan), 1% non-essential amino acids (Nacalai Tesque), and 1% penicillin-streptomycin (Nacalai Tesque). Cells were maintained in a humidified atmosphere with 5% CO_2_ at 37°C. The PARP inhibitors olaparib and talazoparib were purchased from Chemscene LLC (Monmouth Junction, NJ, USA) and MedChem Express (Monmouth Junction, NJ, USA), respectively. FK866 was obtained from Adipogen Life Sciences, and nicotinamide was purchased from Nacalai Tesuque.

### Establishment of BRCA1 KO cancer cells (C1 cells) using CRISPR/Cas9

To establish *BRCA1* KO MIA PaCa-2 cells, we designed a guide RNA (gRNA) sequence for targeting *BRCA1* exon 12 using CHOPCHOP (https://chopchop.cbu.uib.no/). The gRNA sequence was as follows: 5’- tggatttcgcaggtcctcaa-3’. The gRNA was cloned into the plasmid pRP[CRISPR]-Puro-hCas9-U6 by Vector Builder Inc. (Chicago, IL, USA), and plasmids were transfected into MIA PaCa-2 cells using Lipofectamine 3000 (Thermo Fisher Scientific, Tokyo, Japan). Two days after transfection, cells were selected by applying 1 μg/mL puromycin for 3 days, and single clones were isolated. Knockout of *BRCA1* was evaluated by western blot analysis and quantitative RT-PCR.

### Generation of olaparib-resistant cancer cells (C1/OLA cells) from C1 cells

To generate olaparib-resistant cells, C1 cells were treated with 0.75 mM MMS for 1 h and then continuously cultured in the presence of 1–20 μM olaparib for approximately 5 months. Olaparib-resistant clones were maintained in MEM supplemented with 10% FBS, 1% non-essential amino acids, 1% penicillin-streptomycin, and 1 μM olaparib. An isolated single clone#(2–4)9 was designated as C1/OLA cells.

### Western blot analysis

Western blot analysis was performed as described previously [23]. Briefly, cell extracts were prepared using Laemmli buffer. Proteins were separated by SDS-PAGE and transferred onto PVDF membranes. The following antibodies were used for immunoblotting: anti-β-actin (dilution 1:10000; A2228, Sigma-Aldrich), anti-BRCA1 (dilution 1:300; MAB22101, R&D systems (Minneapolis, MN, USA)), anti-BRCA1 (D-9)(dilution 1:100; sc-6954, Santa Cruz Biotechnology (Dallas, TX, USA)), and anti-PARP (dilution 1:2000; #9542, Cell Signaling Technology (Danvers, MA, USA)). Immune complexes were detected using a horseradish peroxidase-linked secondary antibody (Cytiva, Tokyo, Japan), and immunoreactive proteins were visualized using a chemiluminescence kit (Merck, Branchburg, NJ, USA) and the ChemiDoc imaging system (Bio-Rad Laboratories, Hercules, CA, USA). Image quantification was performed using ImageJ software (NIH, Rockville, MD, USA).

### Quantitative RT-PCR (qRT-PCR)

Total RNA was prepared from cells using the High Pure RNA isolation kit (Roche, Basal, Switzerland). Reverse transcription of mRNA into cDNA was performed using the High-Capacity cDNA Reverse Transcription Kit (Thermo Fisher Scientific, Tokyo, Japan). qRT-PCR analysis was performed using SYBR Green with the StepOnePlus Real-Time PCR System (Thermo Fisher Scientific, Tokyo, Japan). Data were normalized to glucuronidase beta (*GUSB*) expression. Primer sequences are listed in Table 1.

**Table 1:** Primer sequences for qRT-PCR.

Target gene	Forward (5’ to 3’)	Revers (5’ to 3’)
*BRCA1*	ATTGCGGGAGGAAAATGGGT	TGAAGGGCCCATAGCAACAG
*PARG*	GTCGAGTCCTGTGCAGAGACC	GGGAGGTGGGAGGAGATGCTA
*NAMPT*	TACAAGTTGCTGCCACCTTATC	GCAAACCTCCACCAGAACC
*NMNAT2*	GTAGTGACCTGCTGGAGTCCTT	ATGATTCGGTCTGTGTCGGCTG
*GUSB*	GCCTGCGTCCCACCTAGAAT	ACATACGGAGCCCCCTTGTC

### Cell proliferation assay

Cell viability was evaluated using the Cell Counting Kit-8 (Dojindo laboratories, Kumamoto, Japan) according to the manufacturer’s instructions. Briefly, cells were seeded onto 96-well plates at a concentration of 1500 cells/well and incubated overnight at 37°C. Then, cells were cultured in the presence of olaparib for 3 days. Nicotinamide was added 1 h before olaparib treatment, as required. Cell viability was determined using the Cell Counting Kit-8, which contains a water-soluble tetrazolium salt (WST-8). Absorbance at 450 nm with a reference of 600 nm was measured using a microplate reader.

### RNA-seq analysis

C1 and C1/OLA cells were seeded at a density of 4 x 10^5^ cells on 10 cm dishes and treated with 10 μM olaparib for 24 h, and total RNA was then extracted from the cells. RNA quality was assessed by DNA Chip Research Inc. (Tokyo, Japan) using an Agilent Technologies 2100 Bioanalyzer, which confirmed an RNA integrity number of 9.0 or higher. Subsequently, an RNA-seq library was generated and RNA-seq analysis was performed by DNA Chip Research Inc. using the TruSeq Stranded mRNA Library Prep Kit (Illumina) and NovaSeq600 (Illumina), respectively. Then, pathway analysis was performed by DNA Chip Research Inc. using PathVisioRPC v1.2 (PathVisio 3).

### Measurement of NAD^+^ levels

Intracellular NAD^+^ levels were determined using the NAD/NADH Assay Kit-WST (Dojindo laboratories) in accordance with the manufacturer’s instructions. Briefly, 3 x 10^5^ cells were harvested and suspended in NAD/NADH Extraction Buffer to lyse the cells. Cell lysates were filtered using MWCO 10K filtration tubes. For measurements of NADH only, half the sample solution was first incubated at 60°C for 1 h to remove NAD^+^. For the measurement of total NAD^+^/NADH and NADH levels, samples were then treated with NAD cycling enzyme to convert NAD^+^ to NADH and incubated in the presence of water-soluble tetrazolium salt at 37°C for 1 h. Sample absorbance at 450 nm was measured using a microplate reader. Intracellular NAD^+^ levels were determined by subtracting NADH levels from total NAD^+^/NADH levels.

### Analysis of *NAMPT* and *NAMPT2* expression in human tumors

The frequency of genetic alterations of *NAMPT* and *NMNAT2* in various cancers was analyzed using the Pan-Cancer Studies dataset from The Cancer Genome Atlas (TCGA) via the cBioPortal for Cancer Genomics (https://www.cbioportal.org/, accessed on 4 May 2023). The Pan-Cancer Studies dataset consists of 10 separate studies with a total of 76639 samples: the MSK-IMPACT Clinical Sequencing Cohort, Metastatic Solid Cancers, MSS Mixed Solid Tumors, the SUMMIT-Neratinib Basket Study, TMB and Immunotherapy, Tumors with TRK fusions, Cancer Therapy and Clonal Hematopoiesis, China Pan-cancer, Pan-cancer analysis of whole genomes, and MSK MetTropism. The UCSC Xena browser (https://Xena.ucsc.edu/) was used to analyze overall survival probability by comparing *NAMPT* mRNA expression levels in pancreatic cancer patients. For Kaplan-Meier analysis, we used 169 samples from TCGA Pancreatic Cancer (PAAD) database.

### Statistical analysis

Statistical analysis was performed using GraphPad Prism10 software (GraphPad Software, CA, USA). *P*-values of <0.05 were considered statistically significant. For each dataset of experiments performed at least three times, the normal distribution was analyzed by the Shapiro-Wilk test. Comparisons between two groups were performed using the Student’s *t*-test for normal distributions and the Mann-Whitney *U* test for non-parametric distributions. For multiple comparisons, ANOVA followed by Tukey’s HSD post hoc analysis was performed for the groups with normal distribution, and Kruskal-Wallis test followed by Dunn’s post hoc analysis was performed for the groups with non-parametric distribution. The log-rank test was used to evaluate the significance of survival analysis between two groups classified based on the expression level of *NAMPT*, and the hazard ratio and its 95% confidence interval for survival analysis was calculated using Mantel-Haenszel method by GraphPad Prism 10 software.

## Results

### Establishment of *BRCA1* knockout pancreatic cancer MIA PaCa-2 cells

**Fig 1:**
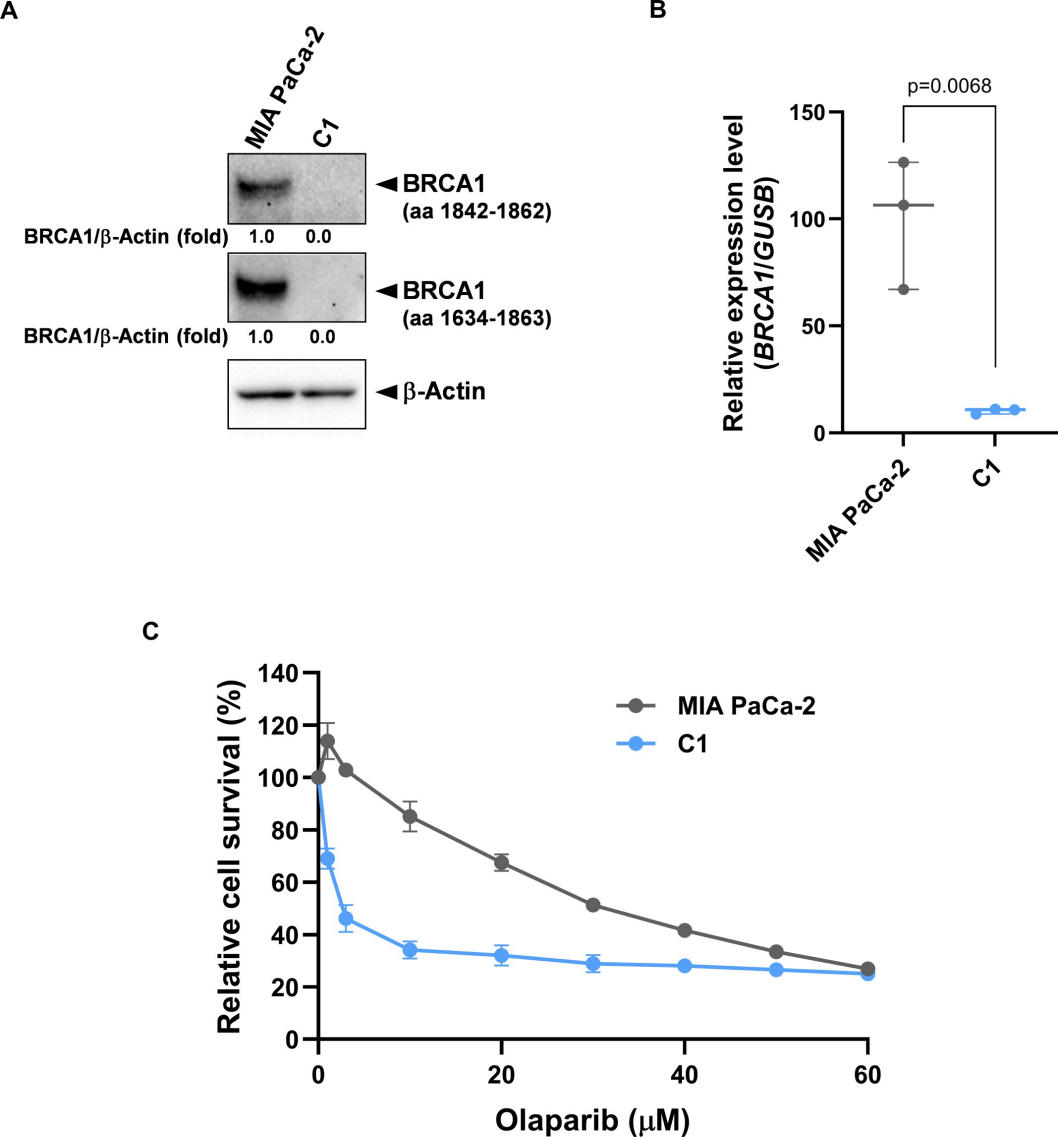
Construction of *BRCA1* KO MIA PaCa-2 cells (C1 cells). (A) Expression level of BRCA1 protein in C1 cells. Whole-cell extracts were analyzed by western blotting using antibodies targeting two BRCA1 epitope sequences (a.a. 1842–1862 and a.a. 1634–1863) (n = 3 independent experiments). (B) The mRNA expression level of *BRCA1* in C1 cells (n = 3 independent experiments). (C) Olaparib sensitivity in C1 cells and MIA PaCa-2 cells. Cells were treated with olaparib at concentrations of 0–60 μM for 3 days. Cell viability was measured by CCK assay. Data are shown as mean ± SEM (n = 3 independent experiments). Statistical comparisons were performed using Student’s *t*-test (B).

To analyze the mechanism of olaparib resistance in pancreatic cancer cells with BRCA1 dysfunction, we first isolated four *BRCA1* KO candidate clones obtained using the CRISPR/Cas9 system. Western blot analysis using two different anti-BRCA1 antibodies showed that BRCA1 was completely downregulated in clone no. (2–4)#9, designated C1 cells in this study (Figs S1A and 1A). In addition, *BRCA1* mRNA levels were decreased to approximately 10% in C1 cells, relative to the levels in the parental cell line (Fig 1B). Cell viability assays showed that C1 cells were more sensitive to olaparib than the other clones (S1B Fig). Moreover, the IC_50_ value for olaparib was approximately 9-fold lower in C1 cells than in MIA PaCa-2 cells (Fig 1C and Table 2). As these results indicated that *BRCA1* was successfully knocked out in C1 cells, cells were used for subsequent analysis.

**Table 2:** IC_50_ and IC_20_ values for olaparib and talazoparib in MIA PaCa-2, C1 and C1/OLA cells.

Cell lines	Olaparib		Talazoparib	
	IC_50_ (μM)	IC_20_ (μM)	IC_50_ (μM)	IC_20_ (μM)
MIA PaCa-2	31.5 ± 2.0	12.8 ± 2.3	ND	ND
C1	3.5 ± 0.49	0.75 ± 0.08	0.018 ± 0.01	0.012 ± 0.002
C1/OLA	> 60	16.5 ± 3.0	> 30	1.2 ± 0.51

ND, not detected

### Generation of the olaparib-resistant C1/OLA cells from *BRCA1* KO MIA PaCa-2 cells

**Fig 2:**
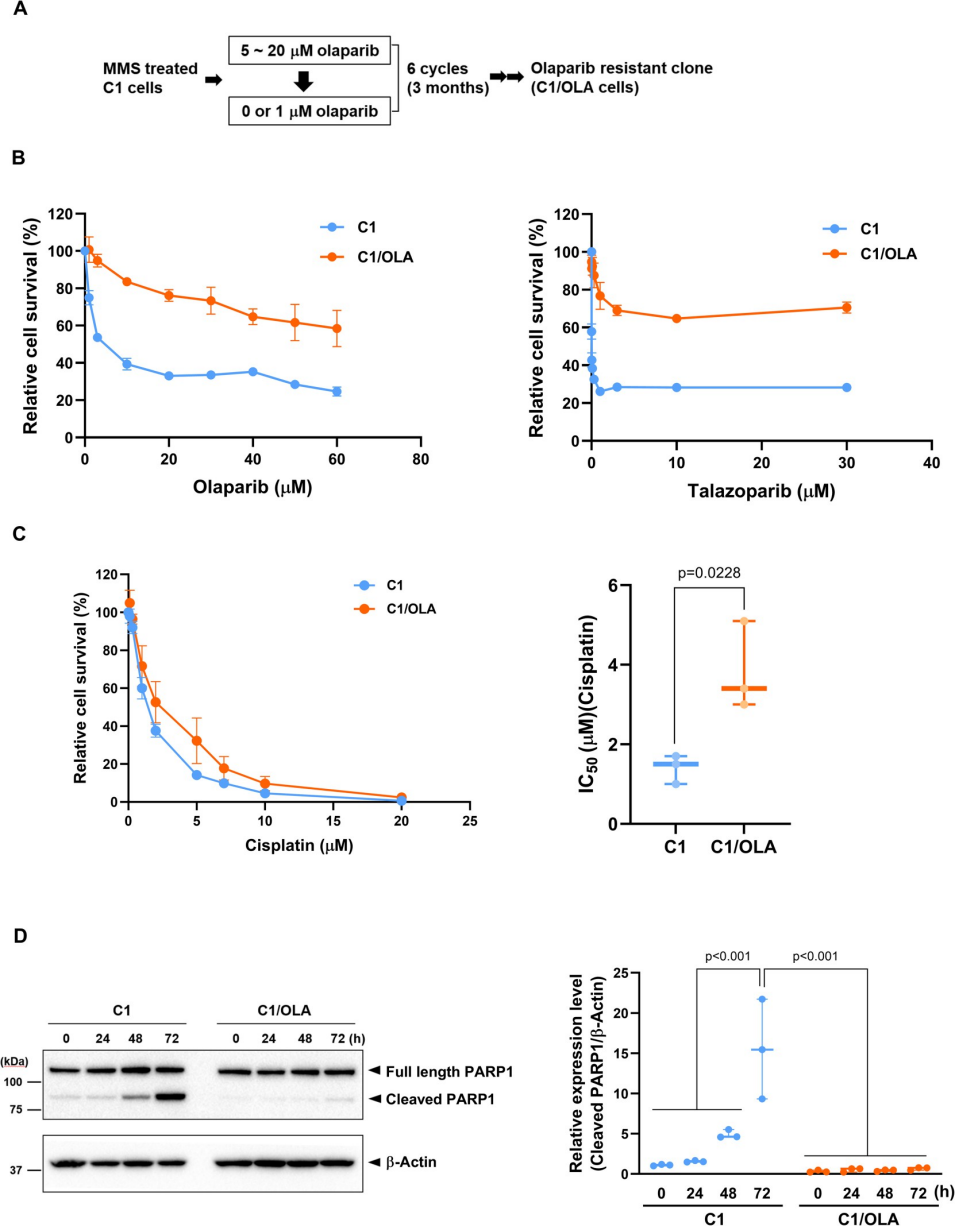
Generation of an olaparib-resistant clone (C1/OLA cells) from C1 cells. (A) Scheme for isolation of an olaparib-resistant clone (C1/OLA). (B) C1/OLA and C1 cells were treated with olaparib (left panel) or talazoparib (right panel) for 3 days, and cell viability was measured by CCK assay (n = 3 independent experiments). (C) C1/OLA and C1 cells were treated with cisplatin for 3 days. Cell viability was measured by CCK assay (Left panel), and IC_50_ values were calculated (Right panel) (n = 3 independent experiments). The IC_50_ values for cisplatin in C1 and C1/OLA cells were 1.4 ± 0.21 μM and 3.8 ± 0.64 μM, respectively. (D) Cells were treated with 10 μM olaparib for 0, 24, 48, and 72 h (n = 3 independent experiments). The expression levels of PARP1 and β-actin proteins were analyzed by western blot analysis (left panel). Cleaved PARP1 levels were normalized to β-actin (right panel). Statistical comparisons were performed using Student’s *t*-test (C) and ANOVA followed by Tukey’s HSD post hoc analysis (D). Data are shown as mean ± SEM (B, C).

To elucidate the mechanism of olaparib resistance, we isolated an olaparib-resistant clone from C1 cells. Methyl methanesulfonate (MMS), the S_N_2 alkylating agent, alkylates DNA with a high yield of N^7^-methylguanine and N^3^-methyladenine [24], thereby inducing mutagenesis of genomic DNA. Glaab *et al*. reported that short-term exposure to MMS increased the mutant frequency in cancer cell lines by approximately two-fold over the spontaneous mutant frequency [25]. Therefore, we used MMS treatment to induce a variety of mutations in the genomic DNA of C1 cells to increase the possibility of identifying novel resistance factors to olaparib. First, to induce mutagenesis in the genomic DNA, we exposed C1 cells to MMS. Then, following continuous exposure of the cells to olaparib, we isolated a single clone of olaparib-resistant cells, designated as C1/OLA cells (Fig 2A). The IC_50_ value for olaparib in C1/OLA cells was more than 17-fold higher than in that of C1 cells (Fig 2B left panel, Table 2). Additionally, C1/OLA cells were also resistant to the PARP inhibitor talazoparib compared with C1 cells (Fig 2B Right panel, Table 2). Interestingly, olaparib-resistant C1/OLA cells also acquired resistance to cisplatin, with an IC_50_ value that was 2.7-fold higher than that of the C1 cells (Fig 2C). At 48 and 72 hours after treatment with 10 μM olaparib, cleaved PARP1 levels were upregulated in C1 cells but not in C1/OLA cells (Fig 2D), suggesting that olaparib treatment induced apoptosis only in C1 cells. These results indicate that we had successfully established a PARP inhibitor-resistant clone.

### Validation of resistance factors in C1/OLA cells

**Fig 3:**
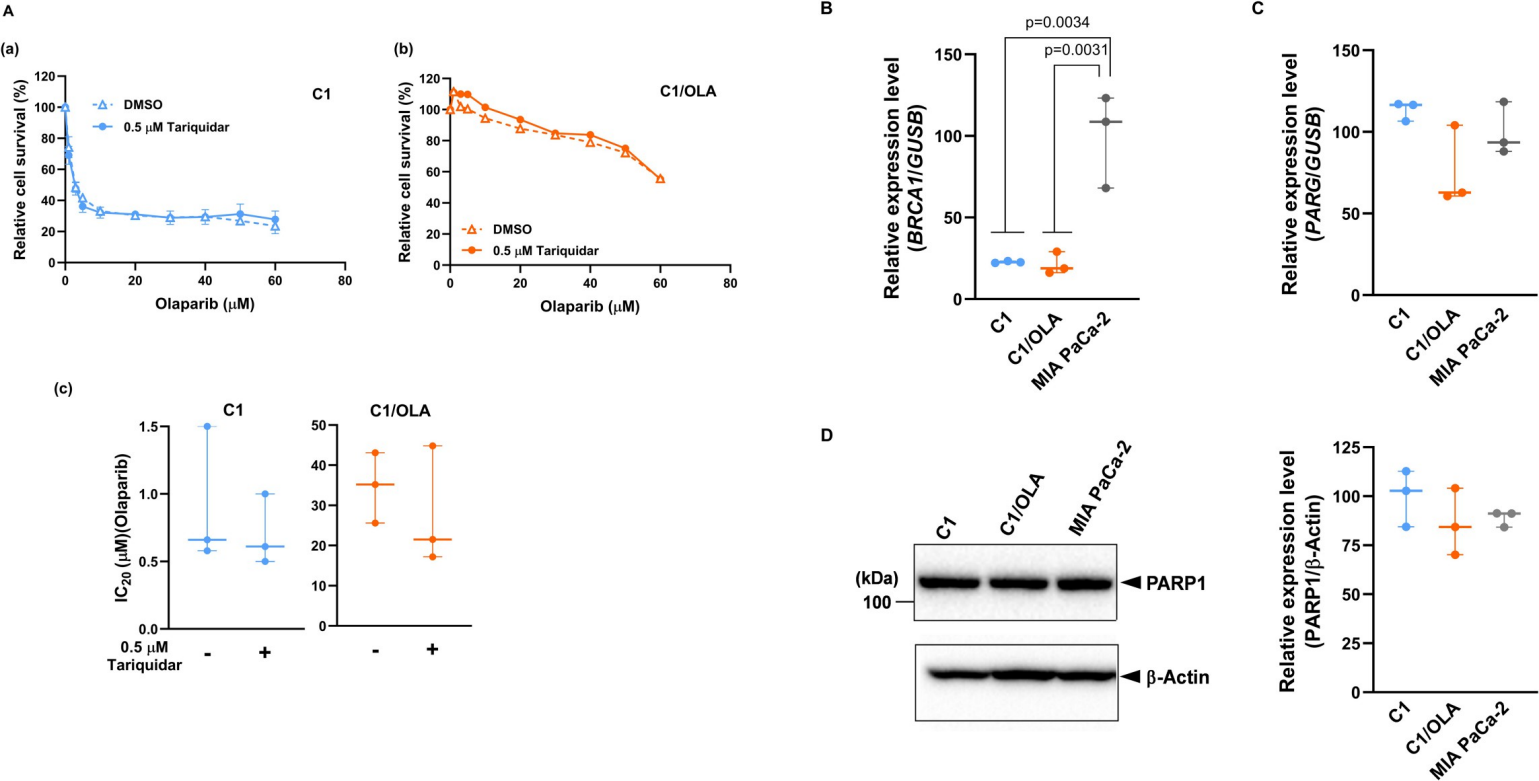
Characterization of olaparib resistance in C1/OLA cells via analysis of the effects of MDR1 and expression levels of *BRCA1*, *PARG*, and PARP1. (A) Cells were treated with olaparib in the absence or presence of 0.5 μM tariquidar for 3 days. Sensitivity to olaparib in C1 cells (a) and C1/OLA cells (b) was analyzed by CCK assay, and IC_20_ values for olaparib were calculated (c). Data are shown as mean ± SEM (n = 3 independent experiments). (B, C) *BRCA1* (B) and *PARG* (C) mRNA levels in C1, C1/OLA and MIA PaCa-2 cells were analyzed by qRT-PCR (n = 3 independent experiments). (D) Expression levels of PARP1 protein in C1, C1/OLA and MIA PaCa-2 cells were analyzed by western blotting (left panel). PARP1 protein levels were normalized to β-actin (right panel) (n = 3 independent experiments). Statistical comparisons were performed using Student’s *t*-test (A(c)), ANOVA followed by Tukey’s HSD post hoc analysis (B, C) and Kruskal-Wallis test followed by Dunn’s post hoc analysis (D).

As previously reported, olaparib resistance can be caused by various factors, including upregulation of the drug efflux pump MDR1, loss of PARG, decreased PARP1 trapping on DNA caused by PARP1 mutation or deletion, and reversion mutations of BRCA1/2 [16, 18]. To elucidate the resistance mechanisms in C1/OLA cells, we validated some of these previously reported resistance factors. First, to examine whether MDR1 contributed to olaparib resistance in C1/OLA cells, we analyzed olaparib sensitivity in the absence or presence of tariquidar, a potent and specific inhibitor of MDR1. The results showed that tariquidar did not affect olaparib sensitivity in either C1/OLA cells or C1 cells, and the IC_20_ values for olaparib were very similar with or without tariquidar in these cells (Fig 3A). In addition, *BRCA1* mRNA remained downregulated to similar levels in C1/OLA and C1 cells (Fig 3B), while *PARG* mRNA levels and PARP1 protein levels were also comparable in the two clones (Fig 3C and 3D). These results suggest that C1/OLA cells may have acquired resistance to olaparib via novel mechanisms.

### Identification of novel mechanisms for olaparib resistance in C1/OLA cells

**Fig 4:**
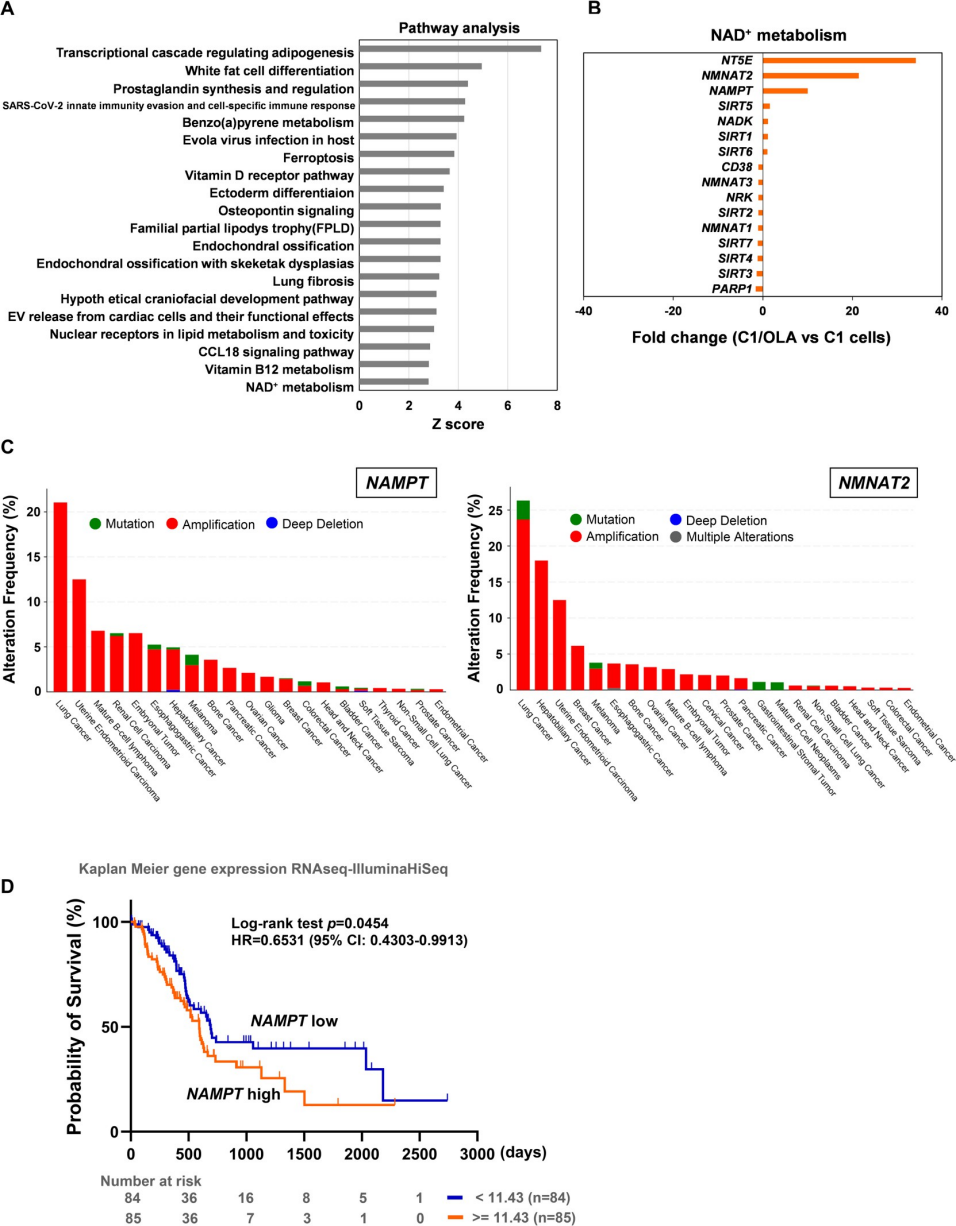
Gene expression profiles for the identification of olaparib resistance mechanisms in C1/OLA cells. (A) Pathway enrichment determined by RNA-seq analysis of olaparib-treated C1/OLA cells vs C1 cells. DEGs increased by more than 2-fold in C1/OLA cells in the RNA-seq data were used for pathway analysis. (B) Pathway analysis showed activation of NAD^+^ metabolism, with upregulation of three DEGs (*NAMPT*, *NMNAT2*, and *NT5E*) among a total of 16 genes in this pathway. (C) Genetic alterations in the frequency of *NAMPT* and *NMNAT2* in tumors were determined from the Pan-Cancer Studies dataset (TCGA database). (D) Overall survival probability comparison in patients showing low and high *NAMPT* expression levels in TCGA Pancreatic Cancer (PAAD) study from TCGA database, analyzed using the UCSC Xena browser. HR, Hazard Ratio; CI, confidence interval.

To elucidate the novel mechanism of resistance to olaparib, we performed RNA-seq analysis in olaparib-treated C1 and C1/OLA cells. Compared with olaparib-treated C1 cells, we detected 2-fold or more upregulation of 1,042 genes and downregulation of 1,259 genes in olaparib-treated C1/OLA cells. Subsequently, pathway analysis showed that differentially expressed genes (DEGs) altered more than 2-fold were associated with the activation of various pathways, including NAD^+^ metabolism (Fig 4A). In this study, we focused on the pathway related to NAD^+^ metabolism because pathway analysis revealed upregulated expression of genes involved in NAD^+^ synthesis: nicotinamide phosphoribosyltransferase (*NAMPT*), nicotinamide nucleotide adenylyltransferase 2 (*NMNAT2*), and 5’-nucleotidase ecto (*NT5E*). No other genes involved in NAD^+^ metabolism were altered by more than two-fold (Fig 4B). *NMNAT2* and *NT5E* were also among the top 40 most highly expressed protein-coding mRNAs. Analysis of TCGA database showed gene amplification of *NAMPT* and *NMNAT2* at relatively high frequency in various types of cancer, including pancreatic, breast, ovarian, and uterine cancers (Fig 4C). Moreover, Kaplan-Meier analysis conducted using the UCSC Xena browser indicated a correlation between high levels of *NAMPT* expression and poor overall survival in pancreatic cancer patients (*p* = 0.0454) (Fig 4D). These results prompted us to further analyze the potential involvement of the NAD^+^ metabolism pathway in olaparib resistance.

**Fig 5:**
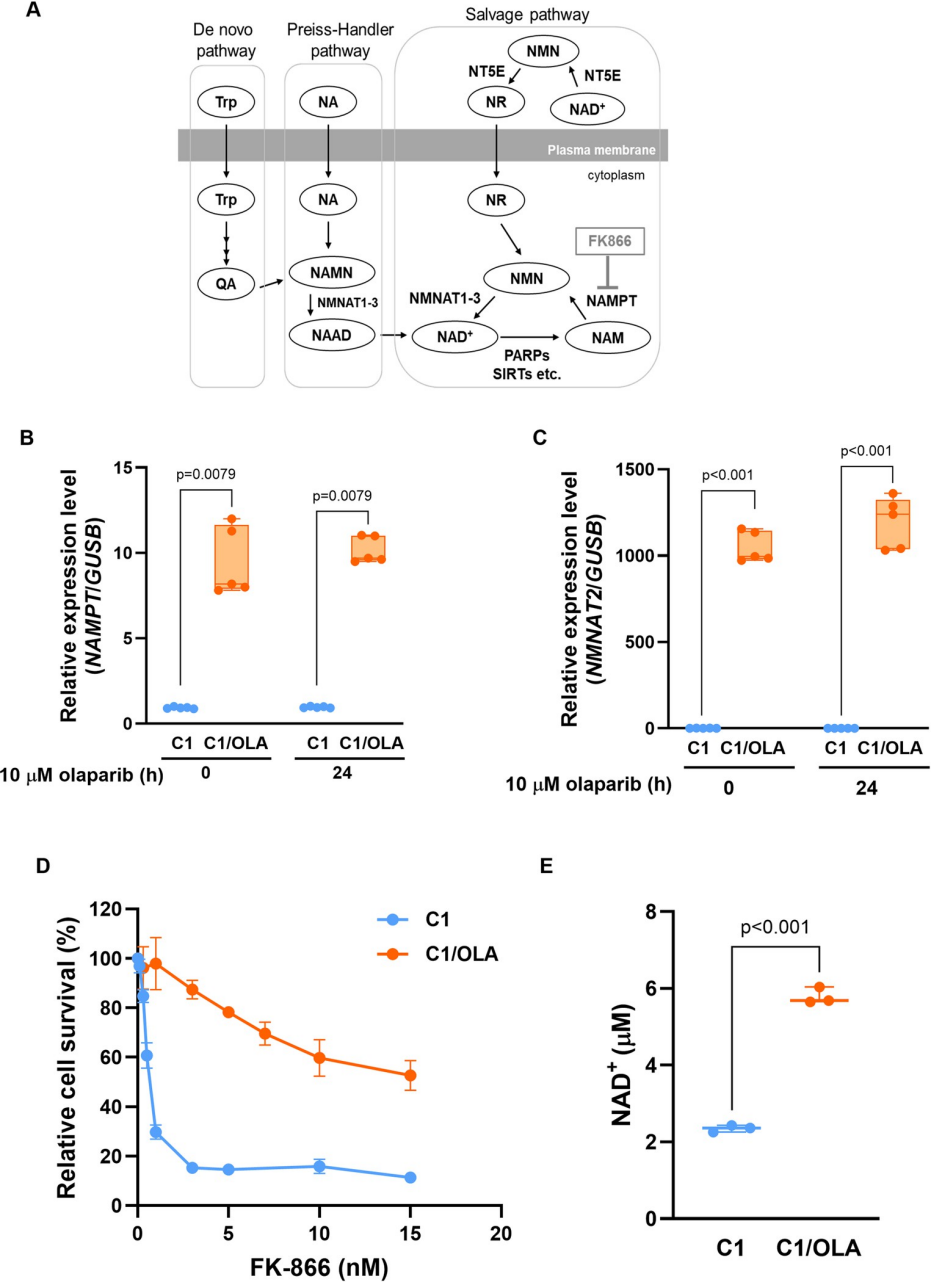
Activation of the NAD^+^ synthesis pathway in C1/OLA cells. (A) NAD^+^ biosynthesis pathway. NAD^+^ synthesis is conducted via the salvage, Preiss-Handler, and de novo pathways. FK866 is a potent inhibitor of NAMPT. (B, C) C1 and C1/OLA cells were treated with or without 10 μM olaparib for 0 and 24 h. *NAMPT* (B) and *NMNAT2* (C) mRNA levels were then determined by qRT-PCR (n = 5 independent experiments). (D) Sensitivity to FK866 in C1 and C1/OLA cells, determined by CCK assay. C1 cells (n = 3 independent experiments), C1/OLA cells (n = 4 independent experiments). (E) Intracellular NAD^+^ levels in C1 and C1/OLA cells. NAD^+^ levels were 2.5 fold higher in C1/OLA cells than in C1 cells (n = 3 independent experiments). Statistical analyses were performed using Mann-Whitney *U*-test (B) and Student’s *t*-test (C, E). Abbreviations: Trp, tryptophan; QA, quinolinic acid; SIRT, sirtuin; NA, nicotinic acid; NR, nicotinamide riboside; NAMN, nicotinic acid mononucleotide; NAAD, nicotinic acid mononucleotide.

NAMPT and NMNAT2 are key factors that synthesize NAD^+^ via the salvage pathway (Fig 5A). In particular, NAMPT is known to be a rate-limiting enzyme in the salvage pathway. Thus, we focused on *NAMPT* and *NMNAT2*, which are directly involved in NAD^+^ synthesis, rather than NT5E, which catalyzes the conversion of extracellular NAD^+^ to nicotinamide mononucleotide (NMN). At 0 and 24 h after treatment with olaparib, *NAMPT* and *NMNAT2* mRNA levels were significantly higher in C1/OLA cells than in C1 cells (Fig 5B and 5C). To evaluate whether the NAD^+^ biosynthesis pathway was activated in C1/OLA cells, we analyzed the effects of the NAMPT inhibitor FK866 on cell viability. We found that the IC_40_ values for FK866 were 19.8 fold higher in C1/OLA cells than in C1 cells, at 10.1 and 0.51 nM, respectively (Fig 5D). Additionally, intracellular NAD^+^ levels were 2.5 fold higher in C1/OLA cells than in C1 cells (Fig 5E). These results suggest activation of the NAD^+^ biosynthesis pathway in C1/OLA cells.

**Fig 6:**
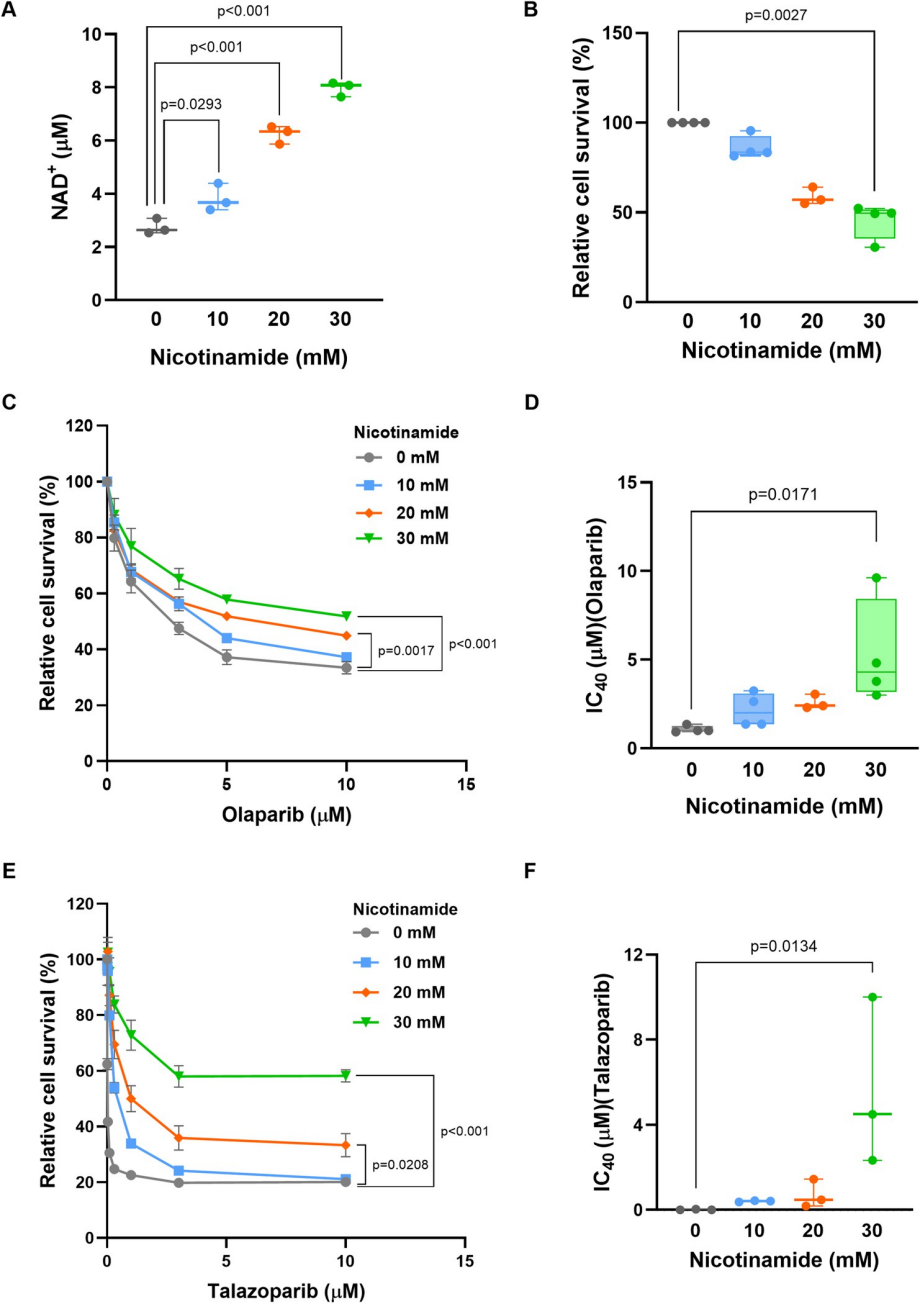
Susceptibility of C1 cells to olaparib following the elevation of intracellular NAD^+^ levels. (A) Intracellular NAD^+^ levels in C1 cells following nicotinamide treatment. Cells were cultured in the presence of 0–30 mM nicotinamide for 24 h, and NAD^+^ levels were then measured (n = 3 independent experiments). NAD^+^ levels increased in a nicotinamide concentration-dependent manner. (B-F) Sensitivity to olaparib in the presence of nicotinamide. C1 cells were treated with 0–10 μM olaparib or 0–10 μM talazoparib in the presence of 0–30 mM nicotinamide. Cell viability was then measured by CCK assay (C, E), and IC_40_ values were calculated (D, F). Cell viability without PARP inhibitor is shown in (B). Treatment of 0, 10 and 30 mM nicotinamide (n = 4 independent experiments), 20 mM nicotinamide (n = 3 independent experiments) in Fig 6B–6F. Statistical comparisons were performed using ANOVA followed by Tukey’s HSD post hoc analysis (A, C, D, E) and Kruskal-Wallis test followed by Dunn’s post hoc analysis (B, F).

Next, to determine whether elevated NAD^+^ levels were involved in the induction of olaparib resistance in C1/OLA cells, we examined whether the increase in intracellular NAD^+^ levels by the addition of nicotinamide would induce resistance to olaparib in C1 cells. The addition of 20 or 30 mM nicotinamide increased NAD^+^ levels by 2.3-fold or 2.9-fold, respectively, compared with levels in untreated C1 cells (Fig 6A). However, while intracellular NAD^+^ levels were elevated in a nicotinamide concentration-dependent manner, nicotinamide treatment also reduced cell viability (Fig 6B). Following the addition of 20 or 30 mM nicotinamide, the levels of intracellular NAD^+^ in C1 cells were similar to those observed in C1/OLA cells. Finally, sensitivity to PARP inhibitor olaparib and talazoparib was evaluated in C1 cells in the absence or presence of nicotinamide (Fig 6C–6F). These results showed that susceptibility to olaparib and talazoparib was decreased in a nicotinamide concentration-dependent manner, respectively (Fig 6C and 6E). Consistent with these results, the IC_40_ values for olaparib and talazoparib were 3.9-fold and 383-fold higher in 30 mM nicotinamide-treated C1 cells than in untreated C1 cells, respectively (Fig 6D and 6F). These findings suggest that elevated intracellular NAD^+^ levels are involved in the induction of resistance to PARP inhibitor in C1/OLA cells.

## Discussion

Recently, the acquisition of drug resistance, including resistance to PARP inhibitors, has been highlighted as a clinical problem that affects prognosis in cancer patients. Various mechanisms of resistance to PARP inhibitors including restoration of HR have been elucidated via studies performed both in vitro and in PARP inhibitor-treated cancer patients [16–21, 26, 27]. However, therapies for overcoming olaparib resistance have not been established, and the factors underlying resistance to PARP inhibitors have not been fully identified. Thus, to explore the novel mechanisms of resistance to PARP inhibitors, we constructed *BRCA1* KO pancreatic cancer cells (C1 cells) and then derived an olaparib-resistant clone (C1/OLA cells) from these cells. C1 cells showed increased susceptibility to olaparib, compared with parental MIA PaCa-2 cells. These results suggest that synthetic lethality was induced in *BRCA1* KO cells under olaparib treatment conditions and predicted decreased HR repair as a result of *BRCA1* defects in C1 cells. Then, robust resistance to both olaparib and talazoparib was induced in C1/OLA cells, indicating the acquisition of resistance to PARP inhibitors by C1/OLA cells via PARP1/2 dysfunction. Thus, we successfully established an olaparib-resistant clone in a background of BRCA1 dysfunction.

Next, we used C1/OLA cells to investigate mechanisms of resistance to olaparib. PARylation cycle enzymes have been reported to be involved in olaparib resistance. Briefly, PARP1 harboring an R591C mutation, which abolishes PARP1 trapping, induced resistance to PARP inhibitor in *BRCA1*-mutated tumors [20]. Olaparib resistance was also induced in *PARP1* KO DT40 cells [28]. And loss of PARG, induced olaparib resistance in *BRCA1*- or *BRCA2*-deleted cancer cells [21]. In this study, our results showed that the expression levels of PARP1 and *PARG* remained unchanged in C1/OLA cells, indicating that olaparib resistance was not associated with aberrant PARylation induced by PARP1 or PARG dysfunction.

Upregulation of the drug effulax pump MDR1 is also involved in olaparib resistance. For example, administration of the MDR1 inhibitor tariquidar with olaparib has been found to suppress tumor growth in *BRCA*-mutated tumors that have acquired resistance to olaparib [19, 29]. Nevertheless, treatment with olaparib and tariquidar did not significantly alter olaparib sensitivity in either C1/OLA or C1 cells. Thus, our results suggest that olaparib efflux by MDR1 makes little contribution to olaparib resistance in C1/OLA cells.

NAD^+^, which is a substrate of PARP1 and PARP2, is synthesized by three pathways: the salvage, de novo, and Preiss-Handler pathways [30]. In this study, we showed that *NAMPT* and *NMNAT2*, which encode enzymes within the NAD^+^ synthesis pathway, were upregulated at the mRNA level in C1/OLA cells, and intracellular NAD^+^ levels were also significantly increased. Additionally, nicotinamide treatment increased intracellular NAD^+^ concentrations in C1 cells and induced resistance to olaparib and talazoparib. These results demonstrated that upregulation of NAD^+^ levels could induce resistance to PARP inhibitors. PARP inhibitors prevent PARylation reactions by competitively binding to the catalytic site (NAD^+^-binding site) of PARP1 and PARP2 [31]. Correspondingly, we suggests that in C1/OLA cells, higher NAD^+^ levels resulting from activation of NAD^+^ synthesis pathway compete with olaparib for binding to PARPs, causing olaparib resistance. In this study, we only used a single cell line, MIA PaCa-2, to analyze resistance mechanisms to PARP inhibitors. Further studies are necessary to clarify whether the observed resistance mechanism is common in other pancreatic cancer cell lines and for other cancer types.

High levels of NAMPT, a rate-limiting enzyme in the salvage pathway, have been reported to induce tumor progression in various cancers such as glioma, melanoma, breast and colon cancers [32–34]. Additionally, multi-omics analysis of PDAC tumors with paired non-cancerous adjacent tissues (NATs) in the Clinical Proteomic Tumor Analysis Consortium (CPTAC) cohort revealed that protein and mRNA expression levels of NAMPT are significantly higher in PDAC tumors than in NATs [35]. In this study, analysis of TCGA database also showed that various tumors, including pancreatic cancers, have frequent gene amplification of *NAMPT* and *NMNAT2*. Kaplan-Meier analysis showed that overall survival was significantly shorter in pancreatic cancer patients with high *NAMPT* expression levels, whereas overall survival was not affected by *NMNAT2* expression. These results suggest that high intracellular NAD^+^ levels caused by the upregulation of *NAMPT* contribute to disease exacerbation in pancreatic cancer and that resistance to PARP inhibitors may be induced in various cancer patients including PDAC by the administration of PARP inhibitors. In future, detailed analysis of the correlation between olaparib resistance and intracellular NAD^+^ levels and/or NAMPT expression should be performed using samples from cancer patients in which olaparib resistance has been induced.

Treatment with olaparib and the NAMPT inhibitor FK866 has been reported to induce synergistic effects in triple-negative breast cancer cells [36]. By contrast, we showed that FK866 and olaparib did not exert synergistic effects on cell viability in C1/OLA cells but, rather, had a similar effect to treatment with olaparib alone (S2 Fig). In cancer cells, the NAD^+^/NADH ratio is higher than in normal cells, which may contribute to accelerated glycolysis and synthesis of serine and fatty acids [30, 37]. Additionally, NAD^+^ is involved in various cellular functions, such as NADP^+^ production, which play a role in redox regulation and nucleotide synthesis to promote cell survival [30, 37]. Our results suggest that increased NAD^+^ may be one of the factors underlying resistance factor to PARP inhibitors. Nonetheless, several complex events associated with activation of NAD^+^ biosynthesis pathway which is favorable for cell survival could potentially contribute to the modulation of PARP inhibitor resistance. Future work will aim to investigate specific factors that could suppress PARP inhibitor resistance.

Olaparib has been approved as maintenance therapy for patients with *BRCA1*/*2*-mutated pancreatic cancer whose disease has not progressed on first-line platinum-based chemotherapy regimens. In this study, olaparib-resistant C1/OLA cells showed greater resistance to cisplatin than C1 cells. In accordance with our results, Wang *et al*. reported that cisplatin resistance was induced by NAMPT overexpression in osteosarcoma cell lines [38]. The overexpression of NAMPT also caused acquired resistance to chemotherapeutic agents such as paclitaxel and fluorouracil [39]. These reports and our findings suggest that NAMPT overexpression in cancer cells may lead to resistance to other chemotherapeutic agents in addition to olaparib, necessitating the future development of therapeutic strategies to overcome multi-drug resistance.

In conclusion, we demonstrated that high NAD^+^ levels caused by activated NAD^+^ biosynthesis induced a novel mechanism of PARP inhibitor resistance in pancreatic cancer cells in vitro. At present, various PARP inhibitors including olaparib, talazoparib, rucaparib, and niraparib have been approved by the Food and Drug Administration, and it is predicted that PARP inhibitor resistance will increase in cancer patients in the future. Our study suggests that *NAMPT* expression and high intracellular NAD^+^ levels may be valuable biomarkers to predict the efficacy of PARP inhibitors. Moreover, these findings may contribute to the development of therapeutic strategies to overcome PARP inhibitor resistance.

## Supporting information

S1 Fig Establishment of *BRCA1* KO clones. (A) Expression level of BRCA1 protein in *BRCA1* KO candidate clones. Whole cell extracts were analyzed by western blot analysis using anti-BRCA1 antibodies with epitope sequences of 1634 to 1863 aa. (B) Sensitivity to olaparib in *BRCA1* KO candidate clones. Cells were treated with 0 to 30 μM olaparib for 3 days, and cell viability was measured by CCK assay.
(TIF)

S2 Fig Olaparib sensitivity in FK866-treated C1/OLA cells. C1/OLA cells were treated with 4.5 nM FK866 and 0–50 μM olaparib, and then cell viability was measured by CCK assay.
(TIF)

S3 Fig Images of original uncropped western blotting. (PPTX)

S1 File Raw data in this study. (XLSX)
