# Supplementary figures and images for: Activated NAD+ biosynthesis pathway induces olaparib resistance in BRCA1 knockout pancreatic cancer cells
Source: PLoS One. 2024 Apr 16;19(4):e0302130. doi: 10.1371/journal.pone.0302130 (PMC11020856; doi:10.1371/journal.pone.0302130)

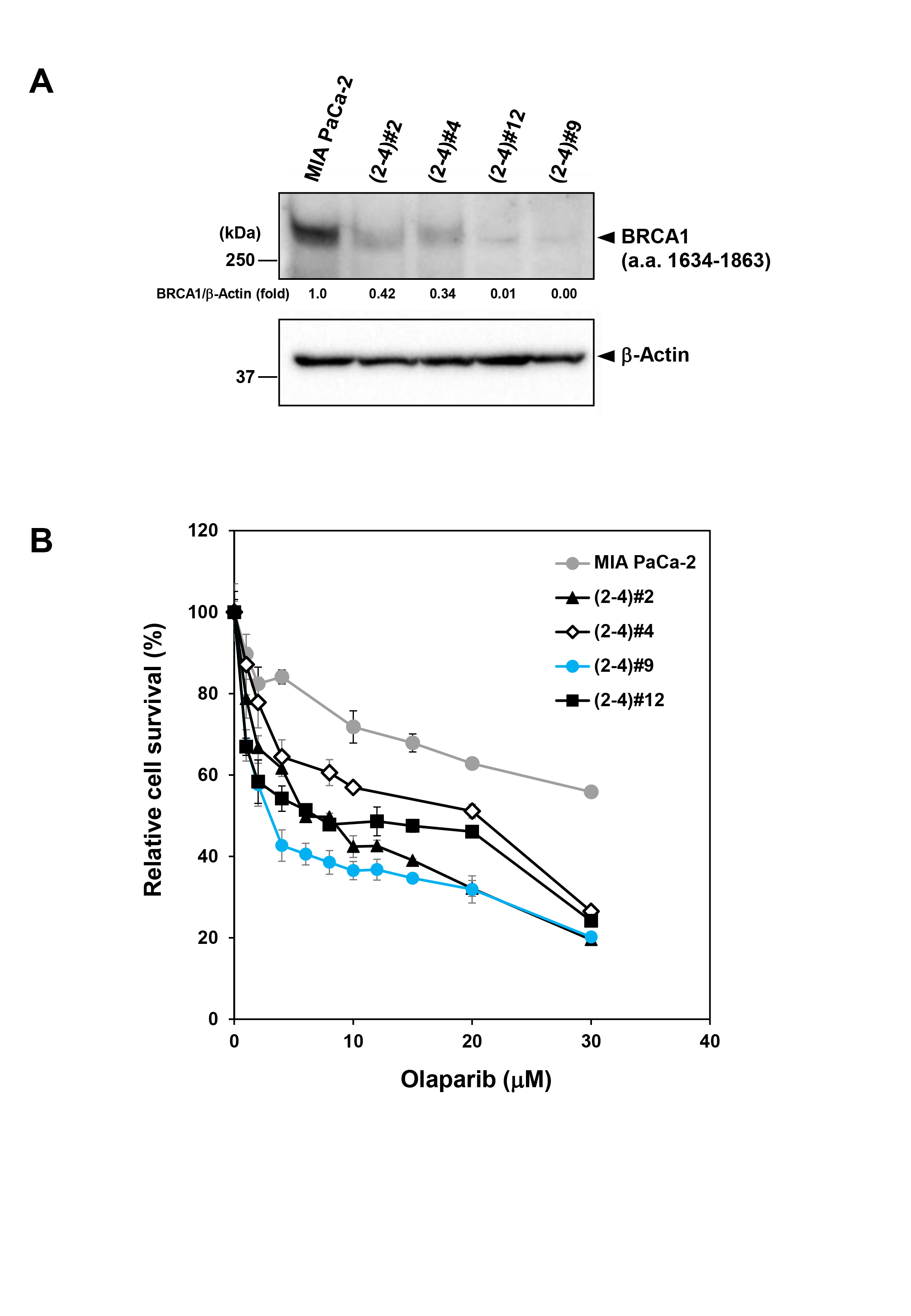

Supplement: S1 Fig — (A) Expression level of BRCA1 protein in BRCA1 KO candidate clones. Whole cell extracts were analyzed by western blot analysis using anti-BRCA1 antibodies with epitope sequences of 1634 to 1863 aa. (B) Sensitivity to olaparib in BRCA1 KO candidate clones. Cells were treated with 0 to 30 μM olaparib for 3 days, and cell viability was measured by CCK assay. (TIF) [file pone.0302130.s001.tif]

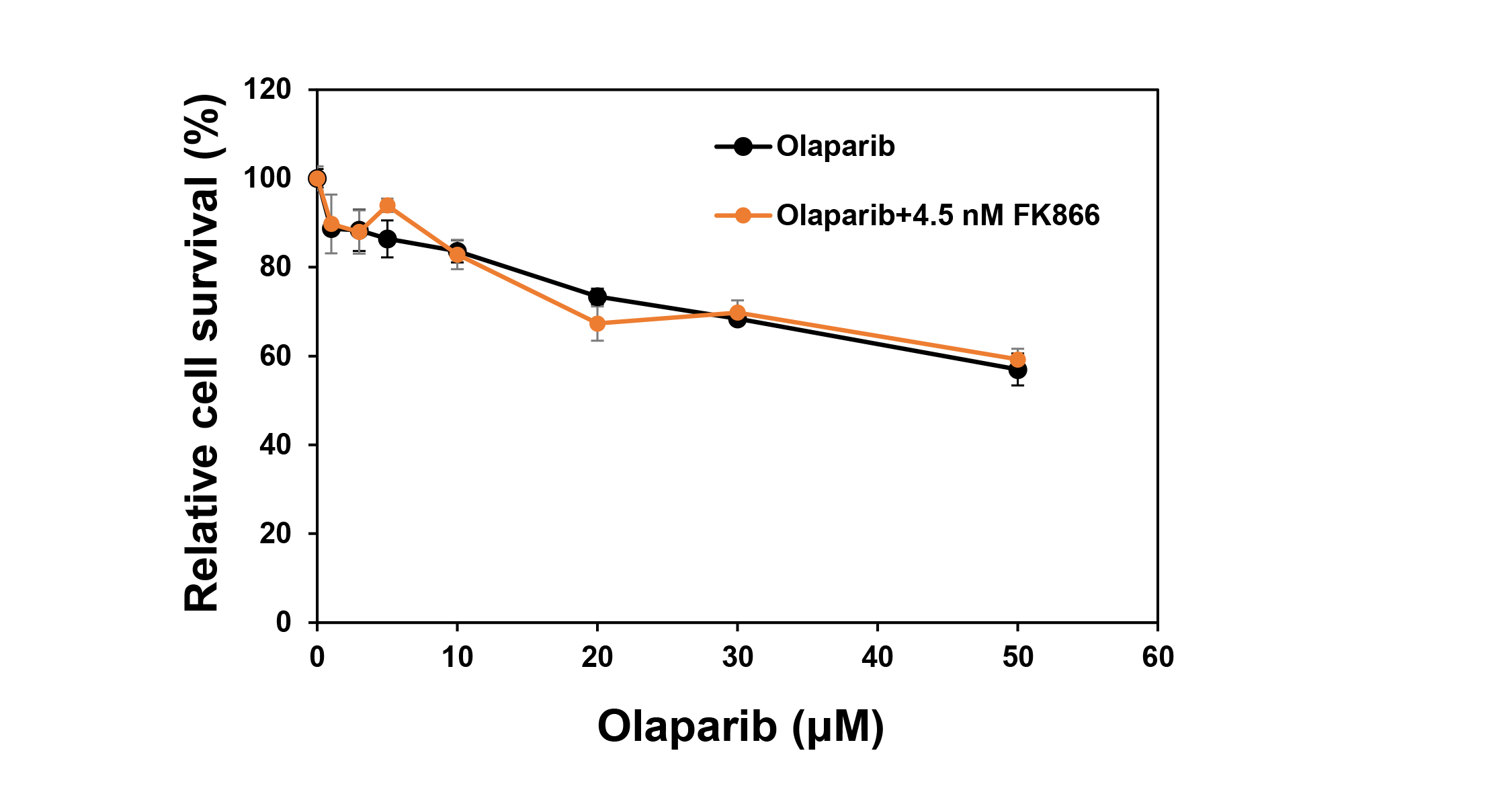

Supplement: S2 Fig — C1/OLA cells were treated with 4.5 nM FK866 and 0–50 μM olaparib, and then cell viability was measured by CCK assay. (TIF) [file pone.0302130.s002.tif]
