# Supplementary material for: Activated NAD+ biosynthesis pathway induces olaparib resistance in BRCA1 knockout pancreatic cancer cells
Source: PLoS One. 2024 Apr 16;19(4):e0302130. doi: 10.1371/journal.pone.0302130 (PMC11020856; doi:10.1371/journal.pone.0302130)
Supplement: S3 Fig — (PPTX) [file pone.0302130.s003.pptx]

## Slide 1
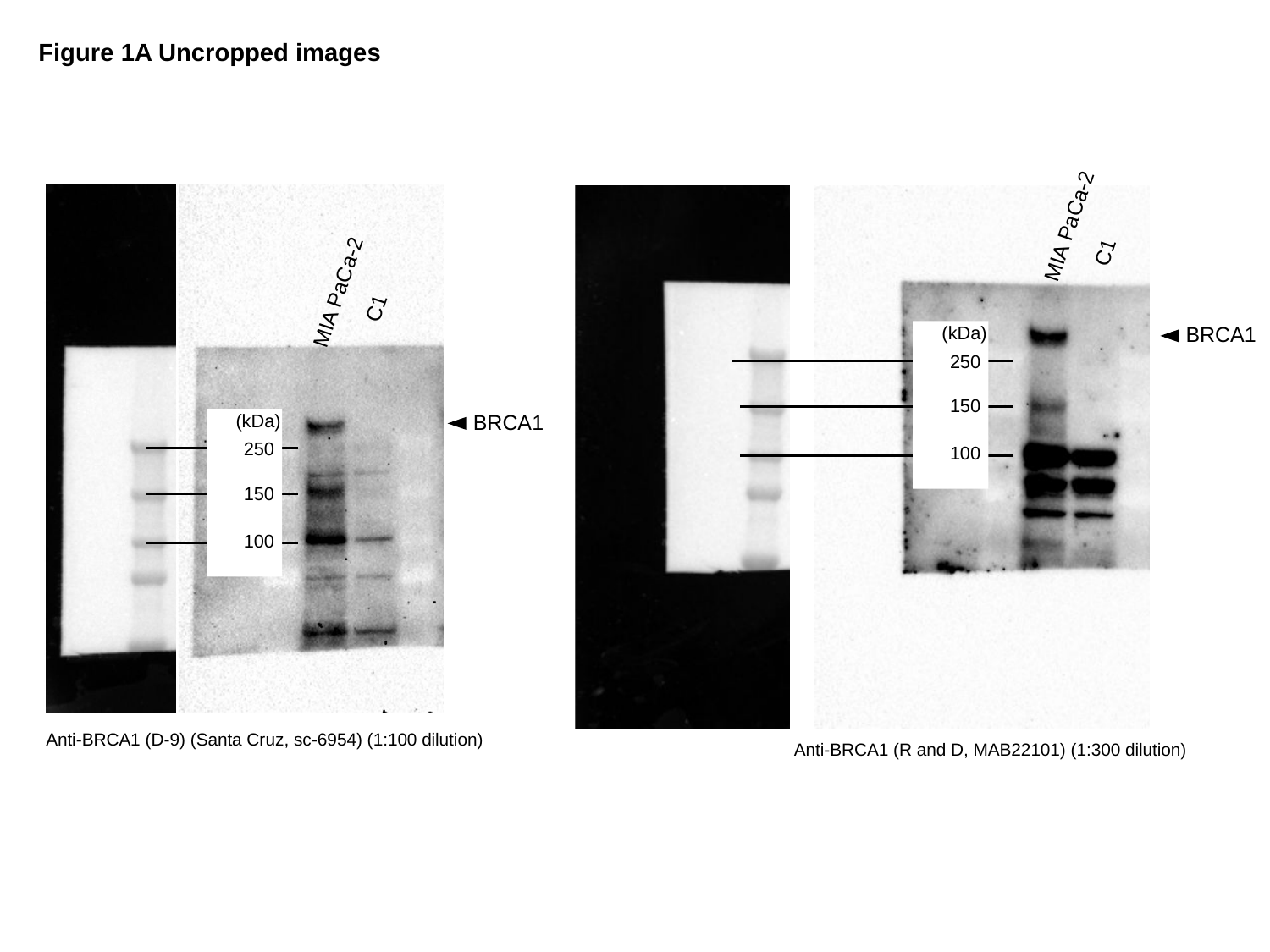

Figure 1A Uncropped images
MIA PaCa-2
C1
(kDa)
BRCA1
250
150
100
Anti-BRCA1 (R and D, MAB22101) (1:300 dilution)
MIA PaCa-2
C1
BRCA1
(kDa)
250
150
100
Anti-BRCA1 (D-9) (Santa Cruz, sc-6954) (1:100 dilution)

## Slide 2
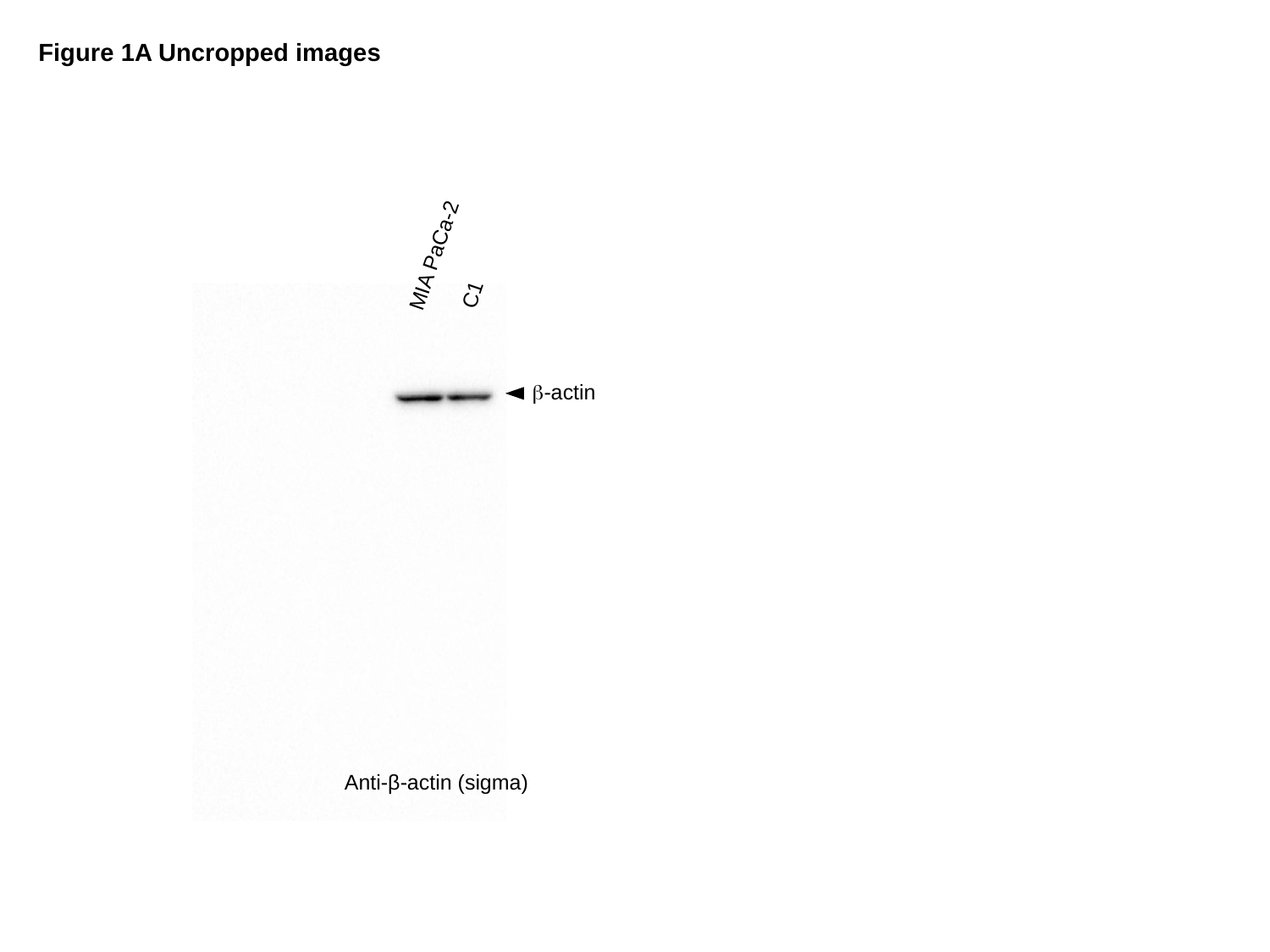

Figure 1A Uncropped images
MIA PaCa-2
C1
b-actin
Anti-β-actin (sigma)

## Slide 3
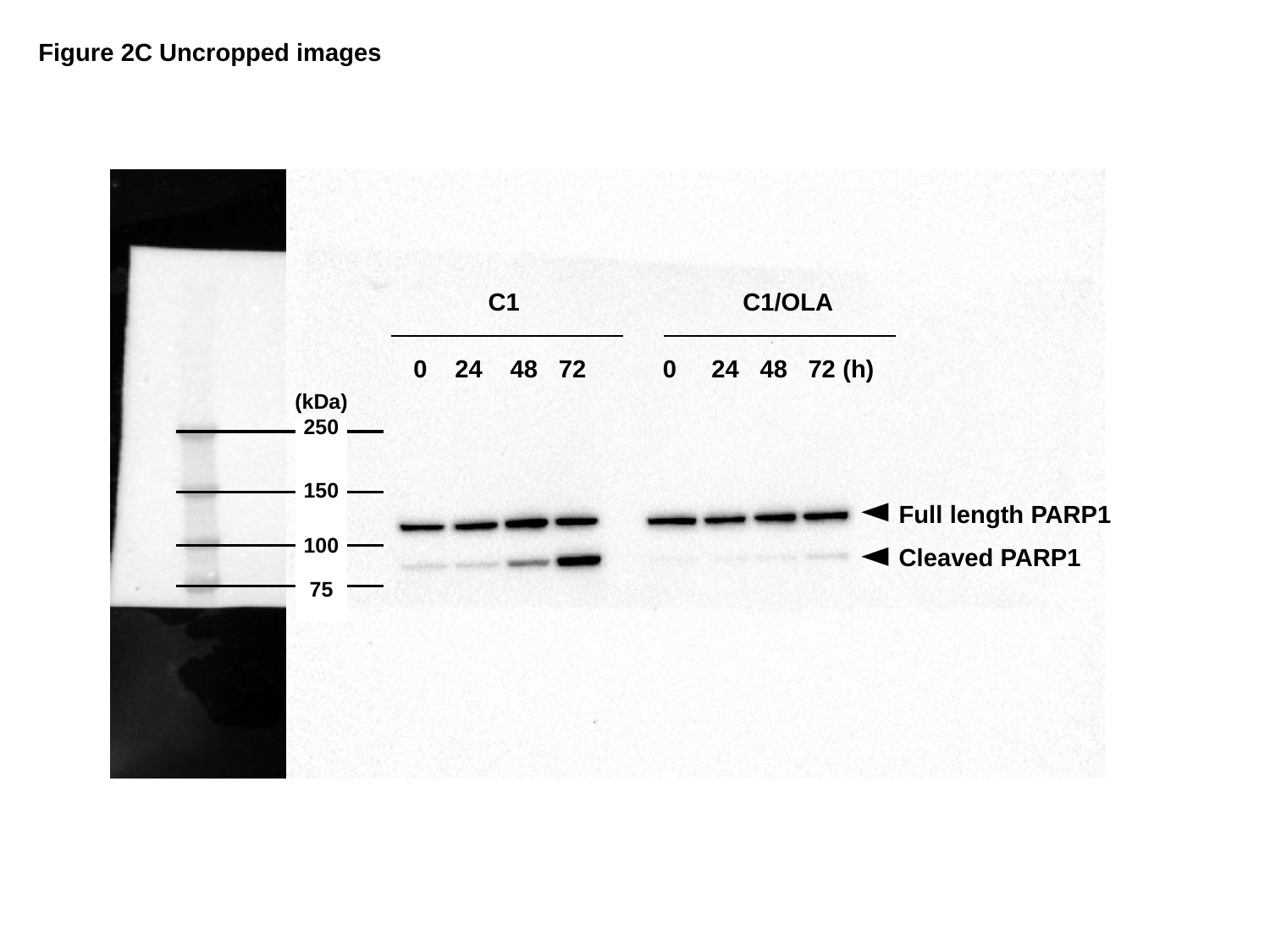

Figure 2C Uncropped images
C1 C1/OLA
0 24 48 72 0 24 48 72 (h)
(kDa)
250
150
100
75
Full length PARP1
Cleaved PARP1

## Slide 4
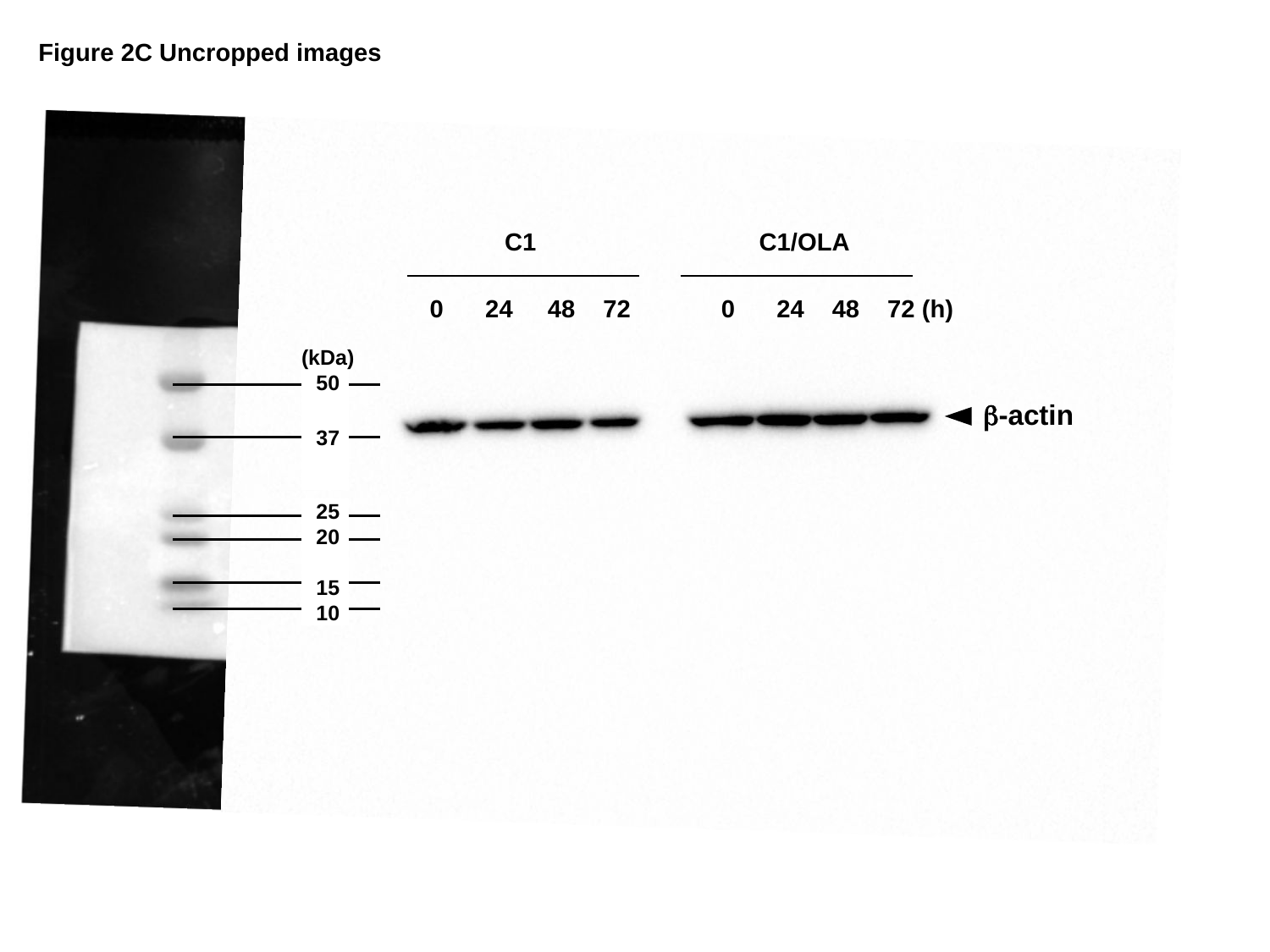

Figure 2C Uncropped images
C1 C1/OLA
0 24 48 72 0 24 48 72 (h)
(kDa)
50
37
25
20
15
10
b-actin

## Slide 5
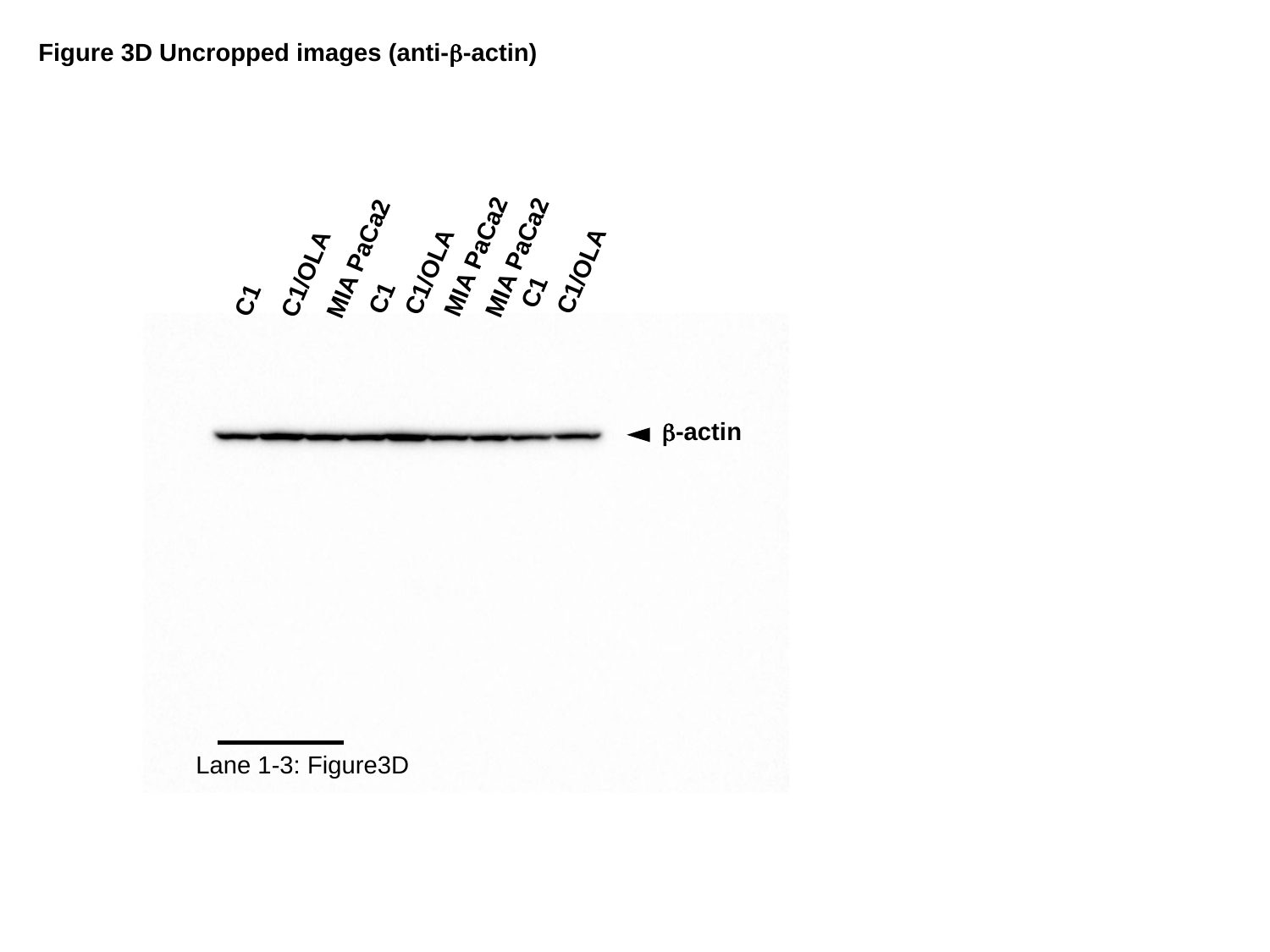

Figure 3D Uncropped images (anti-b-actin)
MIA PaCa2
MIA PaCa2
MIA PaCa2
C1/OLA
C1/OLA
C1/OLA
C1
C1
C1
b-actin
Lane 1-3: Figure3D

## Slide 6
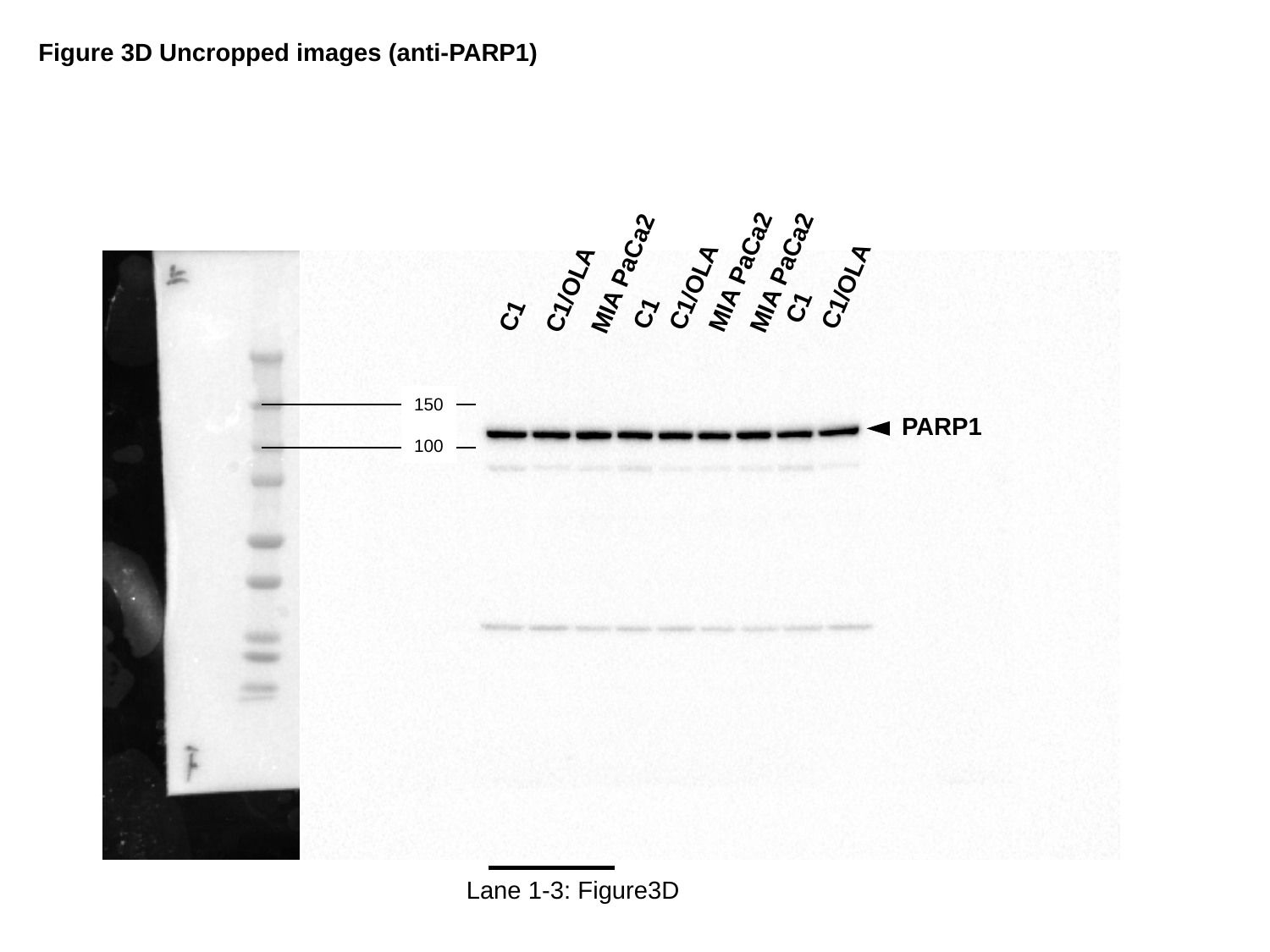

Figure 3D Uncropped images (anti-PARP1)
MIA PaCa2
MIA PaCa2
MIA PaCa2
C1/OLA
C1/OLA
C1/OLA
C1
C1
C1
150
100
PARP1
Lane 1-3: Figure3D
